# Single versus double retrograde intramedullary nail technique for treatment of displaced proximal humeral fractures in children: A retrospective cohort study

**DOI:** 10.1177/18632521241238149

**Published:** 2024-03-18

**Authors:** Eleftheria Samara, Isabella Locatelli, Benjamin Tschopp, Nicolas Lutz, Pierre-Yves Zambelli

**Affiliations:** 1 Pediatric Orthopedic Department, Lausanne Children’s Hospital, Lausanne, Switzerland; 2 Unisanté, Centre universitaire de médecine générale et santé publique, Lausanne, Switzerland

**Keywords:** Proximal humerus fractures, retrograde nail technique

## Abstract

**Background:**

Highly displaced proximal humeral fractures in children with low remodeling potential need to be reduced and fixed. The use of two flexible retrograde nails became the most popular fixation technique due to the excellent functional outcome, the low complication rates, and the possibility of early mobilization. A modified single retrograde technique has been suggested by the authors to address the main disadvantage of this technique, the long operative duration. The aim of this study was to compare these techniques in terms of efficacy, and clinical and radiological outcomes.

**Methods:**

We performed a retrospective, monocentric study. Two groups of patients were defined: One was treated with the standard flexible retrograde double nail technique and the other with the modified single nail technique. The demographic and fracture characteristics were similar in both groups and the postoperative immobilization with a simple sling for 2 weeks. We compared the surgical duration for the initial fixation and hardware removal procedures. The Quick Disabilities of the Arm, Shoulder, and Hand score, the secondary displacement at 1-week follow-up, the radiological union at 6-week follow-up, and the perioperative and short-term complications were also assessed for both groups.

**Results:**

The surgical duration of the initial fixation procedure was significantly shorter in single nail technique group (*p* = 0.005). The percentage of excellent Quick Disabilities of the Arm, Shoulder, and Hand score (0) was similar in the two groups (*p* = 0.98). No secondary displacement was reported for the double nail technique group. In only one patient from the single nail technique group, we detected a secondary displacement at the first week control which did not need reoperation. In both groups, fractures were healed on the 6-week radiologic control. No cases of infection, superficial skin irritation, neurological damage, or complications related to implant removal were reported in both groups.

**Conclusions:**

The single nail technique of fixation proximal humeral fractures in children addresses the disadvantage of long surgical times, described until today, with the double nail technique without compromising the excellent functional and radiological short-term outcomes.

**Level of evidence:**

level III

## Introduction

Proximal humerus fractures (PHFs) in children represent about 2% of all pediatric fractures, with a peak incidence between 11 and 15 years of age.^1,2^ Non-displaced or slightly displaced metaphyseal or physeal PHF in children (Neer-Horowitz (NH) I and II) can be treated conservatively (sling, Desault or Velpeau bandage, cast or hanging cast) independent of the patient’s age.^3–5^ The high remodeling potential of the physis of the proximal humerus is expected to correct axial deviations and arm length discrepancies especially in younger children.^6^ In adolescents, there is still a remodeling potential, and the risk of joint malalignment is low due to the shoulder anatomy, being the joint with three degrees of freedom.^7,8^ Nevertheless, the treatment of NH grade 3 and 4 in adolescents approaching skeletal maturity is debatable in current literature.^9,10^ Several treatment guidelines have been formulated from expert groups for the treatment of PHF based on the patients’ age, the extent of fracture displacement, and comorbidities.^10–14^ Neurovascular injury and open fractures are the only absolute reasons for surgical treatment in skeletally immature patients with PHFs.^10^ Currently, no consensus and no evidence-based guideline have been published concerning treatment options in dependence of patient age, fracture severity, and grade of displacement.^9,15,16^ In recent years, many authors have expanded the relative indications for surgical treatment especially in adolescents approaching skeletal maturity with highly displaced PHF.^9,10,14,16^ In our opinion, this broadening of the indications of surgical treatment is possibly related to observed poor clinical outcomes in older children with highly displaced fractures. Similarly, debatable is the fixation technique used in case of operative treatment.^9,10^ Numerous fixation methods may be used for PHFs: external fixators, free screws, screws, and plate constructs to percutaneous pinning and elastic stable intramedullary nailing (ESIN). The latter two fixation methods tend to be the most popular in adolescent closed PHFs and are both efficient.^2,17–20^ ESIN fixation methods gained popularity over percutaneous pinning—especially in Europe—due to excellent functional outcome with early mobilization and low complication rate. However, ESIN techniques require longer operation time and have higher estimated blood loss, and secondary operation of hardware removal is required, contrary to percutaneous pinning fixation methods.^14,20^ A single retrograde intramedullary nail technique to treat displaced proximal humeral fractures in children was suggested by the authors and proved feasible and efficient.^20^

This study aimed to determine whether there is a significant difference in the surgical duration of the initial fixation procedure between the standard ESIN fixation method of PHF (double nail technique: DNT) and the modified retrograde intramedullary single nail technique (SNT). The surgical duration of the hardware removal procedures was also compared between these two groups. Both durations are considered as efficacy outcomes. Safety outcome measures included the rate of secondary displacement, radiological union at 6-week follow-up, and the Disabilities of the Arm, Shoulder, and Hand (DASH) score at hardware removal time. Our hypothesis is that the SNT method of fixation of PHFs in children is a valid method with shorter surgical duration and with no more complications when compared with the DNT.

## Materials and methods

Between June 2013 and May 2019, we obtained patient details by a review of a regional picture archiving and communications system (PACS), identifying all patients who had received imaging of either shoulder in the trauma unit of the Children University Hospital of Lausanne in Switzerland during this period. These were subsequently reviewed referenced to identify patients who had undergone operative fixation. The inclusion criteria were (1) patients skeletally immature but with low proximal humerus remodeling potential. The remodeling potential was considered low when the lateral half of the proximal humerus physis was thin without or with partial fusion, which corresponds to grade 3 and 4 humeral head ossification grading system as per Li et al. (2) NH grade 3 or grade 4 fracture with angulation of >40 in both coronal and sagittal plan and (3) treatment with closed reduction and single (SNT) or double intramedullary nailing techniques (DNT). Pathological fractures, compound fractures, multiply injured patients, and patients who had undergone surgical fixation in other units were all excluded from the study. Thirty-nine children met the criteria and formed the basis of this study. These patients were then cross-referenced with operation logbooks and Theater Management Systems to ensure data validity, and no loss of patient episode data. Three patients were permanent residents abroad, and we are missing complete follow-up, leaving a population of 36 patients for whom we have complete radiological and clinical records. After approval by the Swiss Ethics Committee (2021-00715), records were reviewed for information regarding sex, fracture type, mechanism of injury, surgical time, complications, duration of primary surgery, duration of the implant removal procedure, and final shoulder range of motion. All children in this cohort were >11 years of chronological age, were classified as stage 3 and 4 humeral head ossification grading, and had a NH grade 3 or grade 4 fracture with angulation of >40. A staff and four fellowship-trained pediatric orthopedic surgeons operated all patients including in this study: From June 2013 to May 2016, the DNT group was the fixation method of use, and from June 2016 to May 2019, the modified retrograde SNT group as described in our previous article^20^ was decided to be used as the only fixation method for patients with PHFs with surgical indication. No patient treated with DNT during this period. Three patients were operated only by staff surgeon in the DNT group and two in the SNT group for insurance reasons. In both groups, a simple sling immobilization was used for 2 weeks postoperatively. Pendulum exercises were immediately initiated after surgery. At 4 weeks postoperatively, active shoulder mobilization according to tolerance was authorized in both groups. Between 3 and 6 months postoperatively, implant removals were suggested and performed to all patients as a day care procedure under general anesthesia. A single pediatric orthopedics fellowship-trained surgeon reviewed standard anteroposterior and Neer radiographic views of all operated patients with both techniques retrospectively at 1, 4, and 6 weeks postoperatively and post-implant removal. The radiographs were evaluated for apposition, angulation, fracture union, and implant-related complications. The French edition of the Quick DASH (shortened version of the DASH questionnaire) score was used for objective clinical assessment at the time of implant removal.

Brief description of the modified retrograde SNT and its difference from the standard DNT as described by the Nancy team initially.

In the SNT, a 3 or 3.5 mm titanium nail is used. The nail is being modified by the surgeon before incision. The tip of the nail is cut to obtain a sharp end, and the nail is bent in a way that the concave site of its curvature will be placed parallel to the calcar. Mini lateral elbow surgical approach is performed about 1–2 cm proximal to the lateral epicondyle to the bone. Using an awl, an entry hole of the lateral humeral cortex is made, and the nail is inserted into the medullary canal using a T-handle. Meticulously and under fluoroscopic control as necessary due to its sharp end, the nail is advanced to the fracture site. Full muscle relaxation is demanded from the responsible anesthesiologist at this stage, and the reduction is performed with traction, progressive abduction, and simultaneous internal rotation. If after fluoroscopic control the reduction is unsatisfactory- due to possible soft tissue interposition on the fracture site- we suggest to perform the opposite maneuver (traction, adduction and extreme external rotation, then rotate the nail in the fracture site to retrieve the interposed soft tissue and afterwards repeat the initial maneuver of progressive abduction-internal rotation. The reduction is verified under fluoroscopic control in both plans (Anteroposterior and Neer views) and considered satisfactory when is anatomic or with no apposition and less than 10° angulation in both plans. The prebent nail is then advanced to the proximal fracture site crossing the remaining proximal humerus physis up to the subchondral bone and turned so that concave site of its curvature is placed parallel to the calcar, as planned. Live fluoroscopic imaging is performed while moving in all directions of the shoulder to verify the fracture stability and extraarticular position of the nail. The nail is then trimmed and buried under the skin.

## Statistical analysis

Variables were summarized using numbers (percentages) for binary and categorical variables and using mean (*SD*) or median (interquartile range) for continuous variables, according to their distributions. Variables were compared between groups using two-sample *t*-test for normally distributed variables, Mann–Whitney–Wilcoxon test for asymmetric continuous variables, and chi-square or Fisher’s exact test for binary or categorical variables, according to the minimum number of subjects in each cell of the contingency table.

## Results

Of the 36 patients included in this study, 17 were fixed with the DNT and 19 with the modified SNT.

## Demographic and fracture characteristics

Patients’ characteristics were similar in the two groups (Table 1). Eight patients (47.1%) in the DNT group and nine patients (47.4%) in the SNT group were girls (*p* = 0.99). Mean age was 13.1 (*SD* = 2.6) years in the DNT group and 12.6 (2.7) years in the SNT group (*p* = 0.57). The mechanism of injury was pedestrian-vehicular accident for two patients in the DNT and three patients in the SNT group, and a fall during a sports-related activity for 15 patients in the DNT and 16 patients in the SNT group (*p* = 1.000). Three patients in DNT and 4 patients in the SNT group had a NH grade 3 fracture, while 14 patients in DNT and 15 patients in the SNT group had a NH grade 4 fracture (*p* = 1.000). Six fractures in DNT and 11 in the SNT group were metaphyseal, while 11 in DNT and 7 in the SNT group were physeal Salter-Harris type II fractures; one physeal Salter-Harris type I fracture was observed in the SNT group (*p* = 0.06). Angulation (°) and apposition (%) were similar in the two groups (Table 1).

**Table 1.** Comparison of individual characteristics and outcomes variables between DNT and SNT groups.

	DNT (*n* = 17)			SNT (*n* = 19)			*p*
**Demographic**							
Age, mean (*SD*)	13.1	2.6		12.6	2.7		0.573^c^
Sex, female, N (%)	8	47.1		9	47.4		0.985^d^
**Fracture characteristics**							
Injury, N (%)							1.000^e^
Pedestrian-vehicular accident	2	11.8		3	15.8		
Sport-related activity	15	81.2		16	84.2		
Neer-Horowitz fracture grade, N (%)							1.000^e^
Grade 3	3	17.6		4	21.1		
Grade 4	14	82.4		15	78.9		
Type of the fracture, N (%)							0.055^e^
Metaphyseal	6	35.3		11	57.9		
Physeal Salter-Harris type II	11	64.7		7	36.8		
Angulation (°), median (IQ)	55^b^	35.5	59	56	45.5	69	0.225^f^
Apposition (%), median (IQ)	0.7^b^	0.6	1	0.8	0.7	1	0.441^f^
**Surgical parameters**							
Titanium nail diameter (mm), median (IQ)	2.5^a^	2.4	2.6	3.5	3	3.5	<0.001^f^
**Outcomes**							
Surgical duration of initial fixation procedure (min), median (IQ)	71	56	90	42	36	58	0.005^f^
Time at hardware removal (months), median (IQ)	5^a^	4	6	4	3	6	0.231^f^
Surgical duration of hardware removal procedure (min), median (IQ)	25^a^	17.8	41	22	16	33.5	0.407^f^
Quick DASH >0, N (%)	11^a^	68.8		13	68.4		0.983^d^

a 1 missing data.

b 2 missing data.

c Two sample *t*-test.

d Chi-square test.

e Fisher’s exact test.

f Mann–Whitney–Wicoxon test.

## Surgical parameters

In both groups, all fractures were reduced in a closed fashion, and only titanium nails of different diameters were used. A median diameter of 2.5 (2.4–3.6) mm was used in the DNT group, while a median diameter of 3.5 (3–4) mm was used in the SNT group (*p* < 0.001; Table 1).

## Efficacy outcomes

**Figure 1.**
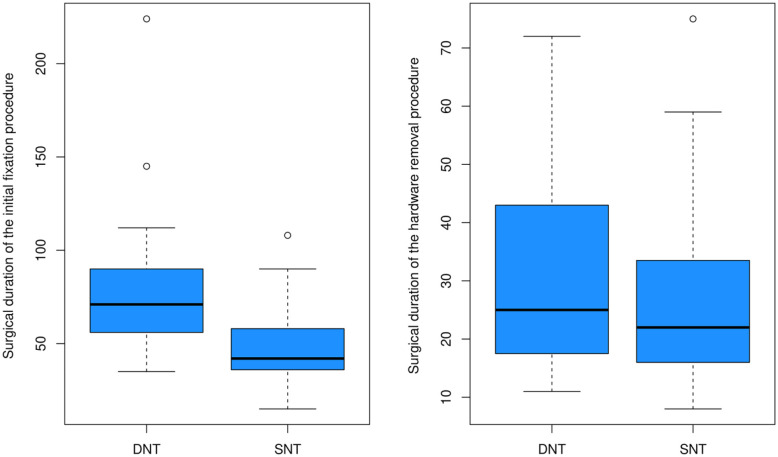
Efficacy outcomes.

Surgical durations: The surgical duration of the initial fixation procedure was significantly shorter in SNT, median time of 42 (36–58) min, versus DNT group, median time of 71 (56–90) min (*p* = 0.005). The hardware removal procedure was conducted after a median of 5 (4–6) months in DNT and 4 (3–6) months in the SNT group (*p* = 0.23), and it required a similar surgical median time of 25 (18–41) min in DNT and 22 (16–34) min in the SNT group (*p* = 0.41; Table 1 and Figure 1).

## Safety outcomes

**Figure 2.**
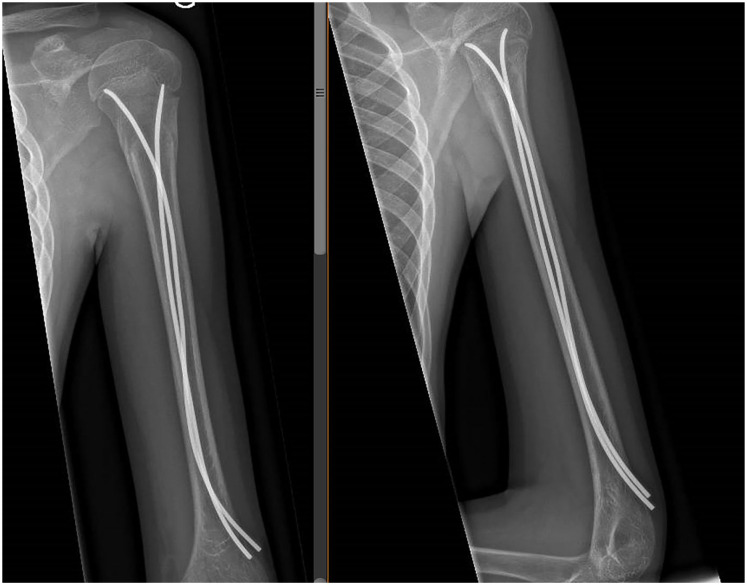
X-ray at 6-week follow-up with the DNT showing radiological union.

**Figure 3.**
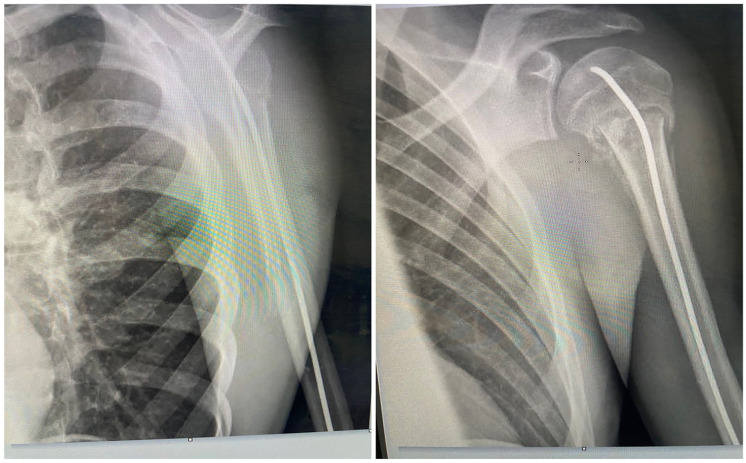
X-ray at 6-week follow-up with the SNT showing radiological union.

- Quick DASH score on the day of implant removal. The percentage of positive Quick DASH score was similar in the two groups, 11 patients (68.8%) in the DNT group and 13 patients (68.4%) in the SNT group (*p* = 0.98), with maximum Quick DASH value of 5 and 4.5 in DNT and SNT groups, respectively (Table 1). The recorded shoulder range of motion in the medical records at the time of implant removal was normal for all patients of both groups.
- Secondary displacement at 1-week follow-up. No secondary displacement was reported for the DNT group. A partial loss of reduction was observed in one case of the SNT group during the first-week control radiograph. For this patient, the calculated apposition was 23% and the angulation was 20° in the AP and Neer views, and the Quick DASH score was zero. This patient did not undergo a reoperation because the displacement was acceptable for his age and potential for remodeling (equivalent to NH grade 2). Critical review of the postoperative radiographs of this patient showed inadequate advancement of the nail in the proximal fragment.
- Radiological union at 6-week follow-up. In both groups, fractures appeared united on the radiographs at the 6-week radiologic control. The fractures were considered united when in orthogonal radiological projections (AP and L views) we observed at least two corticals with callus formation (Figures 2 and 3).

## Complications

In both groups, we had no cases of infection or neurological injuries. No case of elbow stiffness or skin irritation due to protrusion of the nail distally was encountered in both groups. No complications occurred while hardware removals either.

## Discussion

At the moment, there is practice variation among pediatric orthopedic surgeons regarding surgical indication for treatment of PHFs in children. This discordance is due to the inability to estimate accurately the remodeling potential of proximal humerus based on the chronological age. Skeletal maturity is reached at different time points for each individual and is further confounded by their sex, with girls generally being skeletally mature earlier.^9,21^ As Abbot et al.^9^ suggested in their systematic review, a guideline based on combination of radiological fracture displacement and skeletal maturity is required and may be the subject of future prospective clinical trials. However, according to studies published in the last decade, there is a consensus that adolescents treated conservatively for severely displaced fractures present poorer clinical outcome, and as a result, a surgical management may be beneficial.^9,15,22–25^ In our study, indication for surgery was set for children >11 years of chronological age, grade 3 and 4 proximal humerus ossification,^26^ and with a NH grade 4 fracture or a NH grade 3 fracture with angulation of >40, which is consistent to suggestions from other authors.^14,27–30^ Closed reduction under general anesthesia is the mainstay of treatment. Sometimes, the reduction is difficult, especially in 100% apposition cases due to periosteal interposition or interposition of the capsule, long head of biceps tendon or deltoid muscle.^4,5^ In our groups, no patient underwent an open reduction, which is in discordance with previously published articles where open reductions are occasionally needed.^4,5,31,32^

Several fixation methods have been described including pins, elastic nails, screws, plates, or external fixators. Percutaneous pinning and intramedullary nailing are the most common fixation methods, and they result in satisfactory results.^21^ Hohloch et al.^14^ in a meta-analysis reported a preference of intramedullary nailing techniques over percutaneous pinning due to the excellent functional outcome, the low complication rates of the two-nail technique (DNT), and the possibility of early mobilization. The main disadvantages of the intramedullary nail techniques are longer operative times, greater blood loss, and subsequent challenging hardware removal with prolonged operative times.^17,18,33^ To address the disadvantage of prolonged operative time of double intramedullary nail technique, the authors suggested a SNT,^20^ which in this comparative study seems to be a successful fixation method with significantly lower operative time comparing to the DNT and without compromising the fracture stability and subsequently the radiological union and the excellent functional outcomes. All children in the SNT experienced fracture healing at 6 weeks, and painless, complete range of motion after hardware removal.

The DNT of fixation is biomechanically superior as the divergent ends of the nails exert equal forces on both the tension and compression sides of the fracture, whereas a single nail seems to apply an unopposed compression force toward the concavity of its curvature, which is usually parallel to the calcar. In our opinion, the aim of fixation of PHFs in adolescents is to achieve relative stability and maintain the reduction obtained in the operating theater. A construct where the rotation is blocked with a second nail, although obviously more stable, may be not necessary due to the several degrees of freedom of the shoulder and thus its ability to tolerate small rotational malalignment. One nail can act as an internal splint until healing of the fracture and residual remodeling potential of the proximal humerus physis can correct eventual initially radiologically acceptable malalignment.^7,8^

There are limitations on this comparative study that can be addressed in future studies. It is a retrospective analysis of a unique tertiary hospital with limited population due to the restrict volume of patients with surgical indication. This study is, therefore, essentially descriptive, as the small sample size does not allow for adjustment of effects in multivariate models taking into account confounding factors. Prospective multicentric studies are encouraged to validate the efficacy and safety outcomes presented in this study. Five pediatric orthopedic surgeons operated patients in these cohorts, which could also be a limitation, as they have different levels of experience. Moreover, this is a short-period follow-up comparative study. Patients in both groups were followed until complete functional recovery and hardware removal (6 months). Growth arrest or osteonecrosis of the proximal humerus are long-term complications and could not be detected. Longer follow-up study would certainly be of interest.

Despite its limitations, this is a well-designed study since both groups are demographically quite similar, with similar fracture patterns. Some useful clinical conclusions can be provided. Both physeal and metaphyseal fractures can be successfully fixed with a single retrograde intramedullary nail technique. Short immobilization regimens are also sufficient with the SNT as for DNT, without any prolongation.

This is also the first study that demonstrates that a single intramedullary retrograde fixation technique of PHF in children has reduced surgical times, comparing to the DNT without compromising the excellent functional and radiological short-term outcomes.

## Supplemental Material

sj-pdf-1-cho-10.1177_18632521241238149 – Supplemental material for Single versus double retrograde intramedullary nail technique for treatment of displaced proximal humeral fractures in children: A retrospective cohort study Supplemental material, sj-pdf-1-cho-10.1177_18632521241238149 for Single versus double retrograde intramedullary nail technique for treatment of displaced proximal humeral fractures in children: A retrospective cohort study by Eleftheria Samara, Isabella Locatelli, Benjamin Tschopp, Nicolas Lutz and Pierre-Yves Zambelli in Journal of Children’s Orthopaedics
